# Comparative Analysis of Automated and Handheld Breast Ultrasound Findings for Small (≤1 cm) Breast Cancers Based on BI-RADS Category

**DOI:** 10.3390/diagnostics15020212

**Published:** 2025-01-17

**Authors:** Han Song Mun, Eun Young Ko, Boo-Kyung Han, Eun Sook Ko, Ji Soo Choi, Haejung Kim, Myoung Kyoung Kim, Jieun Kim

**Affiliations:** 1 Department of Radiology, Seoul St. Mary’s Hospital, College of Medicine, The Catholic University of Korea, Seoul 06591, Republic of Korea; im_hsm@catholic.ac.kr; 2 Department of Radiology, Center for Imaging Science, Samsung Medical Center, Sungkyunkwan University School of Medicine, Seoul 06351, Republic of Korea; bkhan@skku.edu (B.-K.H.); mathilda0330@gmail.com (E.S.K.); jisoo.choi@samsung.com (J.S.C.); hjk220@naver.com (H.K.); myoungkk@gmail.com (M.K.K.); 3 Department of Radiology, Inje University Haeundae Paik Hospital, Busan 48108, Republic of Korea; dmsdl0625@naver.com

**Keywords:** breast cancer, cancer screening, ultrasound, automated breast ultrasound

## Abstract

**Objectives**: This study aimed to compare ultrasound (US) findings between automated and handheld breast ultrasound (ABUS and HHUS, respectively) in small breast cancers, based on the breast imaging reporting and data system (BI-RADS) category. **Methods**: We included 51 women (mean age: 52 years; range: 39–66 years) with breast cancer (invasive or DCIS), all of whom underwent both ABUS and HHUS. Patients with tumors measuring ≤1 cm on either modality were enrolled. Two breast radiologists retrospectively evaluated multiple imaging features, including shape, orientation, margin, echo pattern, and posterior characteristics and assigned BI-RADS categories. Lesion sizes were compared between US and pathological findings. Statistical analyses were performed using Bowker’s test of symmetry, a paired *t*-test, and a cumulative link mixed model. **Results**: ABUS assigned lower BI-RADS categories than HHUS while still maintaining malignancy suspicion in categories 4A or higher (54.8% consistent with HHUS; 37.3% downcategorized in ABUS, *p* = 0.005). While ABUS demonstrated less aggressive margins in some cases (61.3% consistent with HHUS; 25.8% showing fewer suspicious margins in ABUS), this difference was not statistically significant (*p* = 0.221). Similarly, ABUS exhibited slightly greater height–width ratios compared to HHUS (median, interquartile range: 0.98, 0.7–1.12 vs. 0.86, 0.74–1.10, *p* = 0.166). No significant differences were observed in other US findings or tumor sizes between the two modalities (all *p* > 0.05). **Conclusions**: Small breast cancers exhibited suspicious US features on both ABUS and HHUS, yet they were assigned lower BI-RADS assessment categories on ABUS compared to HHUS. Therefore, when conducting breast cancer screening with ABUS, it is important to remain attentive to even subtle suspicious findings, and active consideration for biopsy may be warranted.

## 1. Introduction

Automated breast ultrasound (ABUS) is a specialized breast US technique that automatically scans the entire breast in transverse sections using a wide transducer. Volume data from ABUS can provide three-dimensional breast reconstructions and simultaneously obtain coronal and sagittal images [1]. This system received U.S. Food and Drug Administration clearance as a supplemental modality in addition to mammography screening in 2008 [2].

Mammography has known limitations in detecting small breast cancers, especially in women with dense breast tissue. Dense tissue can mask tumors on mammograms, reducing the sensitivity of 2D mammography [3,4,5]. This is a key reason why complementary imaging techniques like ABUS and handheld breast ultrasound (HHUS) are used to improve detection accuracy in such cases. Several prospective studies have indicated that the supplementation of mammography with ABUS screening yields comparable favorable outcomes to HHUS screening. These outcomes include the additional detection of cancer, ranging from 1.9 to 2.4 per 1000 screened women with heterogeneously dense or extremely dense breasts. The majority of these detected cancers were invasive, and an acceptable increase in recall rates was observed [6,7]. Despite technical disparities between ABUS and HHUS, prior research has not identified significant differences in diagnostic performance for breast cancer or in inter- and intra-reader agreement between these techniques [8,9,10]. ABUS less frequently identifies smaller and benign lesions, resulting in a lower final assessment category. Nevertheless, it consistently detects all malignant lesions identified on HHUS [11]. However, there is limited research directly comparing lesion sizes in final pathological reports after breast cancer surgery using US measurements from both ABUS and HHUS [12,13].

Over time, the prevalence of small breast cancers has increased due to evolving screening strategies [14]. The most frequently diagnosed invasive breast cancers in developed countries are T1 tumors, encompassing T1a (≤5 mm), T1b (>0.5 but ≤1 cm), and T1c (>1 but ≤2 cm) tumors [15,16]. These smaller tumors exhibit favorable prognoses, with cancer-specific survival rates as high as 90% or 95% [17,18,19].

Given the limitations of mammography, the study focused on small breast cancers (≤1 cm), as this size range presents unique diagnostic challenges, especially in patients with dense breast tissue. Complementary imaging modalities such as ABUS and HHUS are critical for improving diagnostic accuracy in these cases. Limited information exists about the clinical application of ABUS assessments using breast imaging reporting and data system (BI-RADS) categories, especially for small breast masses (≤1 cm), and whether the corresponding results align with those obtained through HHUS [10]. Accurate characterization of suspicious breast masses is essential in clinical practice as it provides crucial information for interpreting biopsy results in conformance with imaging findings. Discrepancies in BI-RADS categorization, depending on the type of US used, can significantly impact the quality of patient care, leading to early cancer diagnosis and avoidance of unnecessary biopsies.

Therefore, this study aimed to compare the US findings of ABUS and HHUS for small (≤1 cm) breast cancers based on the BI-RADS category.

## 2. Materials and Methods

### 2.1. Study Subject

The data for this study were derived from a subset of patients included in the registry of a prospective study (NCT04607473), which aimed to assess the diagnostic performance of ABUS in the preoperative evaluation of patients with early-stage breast cancer. Patients were enrolled between October 2019 and December 2020 and were included if they had newly detected, biopsy-confirmed breast cancer and had voluntarily consented to participate in the study.

Patients were not eligible for participation if they had undergone neoadjuvant chemotherapy or any form of breast cancer treatment prior to the US examinations, or if they had previously undergone breast surgery or radiation therapy, even if they wished to participate in the study.

Both ABUS and HHUS examinations were performed on the same day or within one week of each other, and breast cancer surgery was performed within one month of the US examination.

A total of 499 patients were enrolled in the prospective study, and of these, only patients with breast cancer measuring ≤1 cm on either ABUS or HHUS were included in our analysis. Ultimately, 51 patients with 51 breast masses were analyzed in this study.

The results of the prospective study (NCT04607473) have not yet been published.

### 2.2. US Examinations

#### 2.2.1. ABUS

All ABUS examinations were conducted using the Invenia ABUS system (Reverse Curve™ Ultra-broadband Transducer, GE Healthcare, Sunnyvale, CA, USA) equipped with an automated 6–15 MHz, 15.3 cm wide-field view transducer. During the examination, the patients were placed in the supine position with a sponge placed beneath the shoulders to ensure an even distribution of the breast tissue. The scanning procedure involved bilateral whole-breast scans encompassing the anteroposterior, lateral, and medial views. Additional scans were performed if the basic scans did not adequately cover the entire breast. The scanning depth ranged from 4 to 5 cm and spanned from the skin to the chest wall muscles, including the pectoralis major and intercostal muscles. Following image acquisition, post-processing algorithms were applied to enhance the diagnostic information quality based on nipple location. The obtained volume data were automatically transmitted from the ABUS scanner to a review workstation. Subsequently, the volume data were assessed in the axial, sagittal, and coronal planes on a review workstation, with a 0.5-mm slice interval.

#### 2.2.2. HHUS

Bilateral whole-breast HHUS was conducted by one of the five breast imaging radiologists, each with 10–25 years of experience in breast US. They utilized an IU-22 unit with a 5–12 MHz linear transducer (Philips Medical Systems, Bothell, WA, USA), an RS80A system with a 3–12 MHz linear transducer (Samsung Medison Co., Ltd., Seoul, Republic of Korea), or an Aixplorer System with a 15–4 MHz linear transducer (Supersonic Imagine, Aix-en-Provence, France). All US examinations were performed with the patient in the supine position with both arms elevated. The scanning depth ranged from 3 to 5 cm, covering the skin to the chest wall muscle. A focal zone band was placed at the center of the breast parenchyma. Mild manual compression with a transducer was applied during the examination. Each breast was systematically examined in four quadrants, along with the subareolar area and both axillae, and the findings were recorded for each patient.

### 2.3. Analysis of US Features and Pathologic Data

For comparative analysis, ABUS and HHUS images of breast cancers were jointly reviewed and re-assessed by two board-certified radiologists (H.S.M. and E.Y.K.) with 10 and 19 years of experience, respectively, reaching decisions by consensus. The reviewers analyzed the US findings without access to information about the patient’s age, family history, or findings from other imaging modalities, such as mammography and breast magnetic resonance imaging. However, they were aware that the US images under review were obtained from preoperative studies of patients with breast cancer.

To ensure consistency, the BI-RADS lexicons were applied uniformly to both ABUS and HHUS images, facilitating an unbiased comparison [20]. ABUS findings were analyzed using multiplanar images in three different planes (axial, sagittal, and coronal) with volume data on a dedicated Invenia ABUS review workstation (GE Healthcare, Chicago, IL, USA). For HHUS image analysis, the radiologists were blinded to Doppler and elastography data, relying solely on B-mode static images. To ensure unbiased evaluation, a separate research folder was created on the PACS system (Centricity 2.0, GE Healthcare, Chicago, IL, USA) exclusively for reviewing HHUS images. One of the co-authors (J.S.C.), who was not involved in the image analysis process, uploaded the B-mode static images of HHUS into this folder. To minimize recall bias and ensure independent evaluations, a one-month interval was maintained between the review and assessment of ABUS and HHUS findings.

All 51 small breast cancers included in this study were masses. We analyzed the US features of the lesions, including the shape (oval, round, or irregular), orientation (height–width ratio), margin (circumscribed, microlobulated, indistinct/angular, or spiculated), echo pattern (hyperechoic, isoechoic, heterogeneous echoic, hypoechoic, complex solid, or cystic), and posterior features (no feature, enhancement, shadowing, or combined pattern). Furthermore, they re-assigned the BI-RADS final assessment categories just on the basis of US findings that suggested the risk of malignancy without considering that they were all biopsy-proven cancers according to the BI-RADS assessment category [20]. The pathological data, including operation type, histological diagnosis of the tumors, and tumor size, were reviewed. Pathological tumor size was used as the gold standard for size comparison.

### 2.4. Statistical Analysis

We examined the symmetry of categorical data related to breast mass characteristics, including the shape, margin, echo pattern, and posterior features observed on ABUS and HHUS, using Bowker’s test of symmetry. Paired *t*-tests were used to compare the mass orientation (height–width ratio) and size measurements obtained using ABUS and HHUS. The size measurements were then assessed individually for each modality (ABUS vs. pathology and HHUS vs. pathology). Differences between the imaging modalities (ABUS and HHUS) and their corresponding pathological results were also evaluated using paired *t*-tests. Considering that the final BI-RADS assessment categories for ABUS and HHUS represented ordered categorical response variables, we analyzed them using a cumulative link mixed model (CLMM).

All statistical analyses were performed using SAS version 9.4 (SAS Institute, Cary, NC) and R 4.4.0 (Vienna, Austria; http://www.R-project.org, accessed on 16 January 2024). Two-tailed *p*-values < 0.05 were considered statistically significant.

## 3. Results

The mean age of the enrolled 51 patients was 52.1 ± 7.1 years (range, 39–66 years). Among the 51 patients, 44 underwent breast-conserving surgery, while 7 had total mastectomies. The final pathological reports revealed invasive ductal carcinoma in 41 patients (80.4%), ductal carcinoma in situ in 7 patients (13.7%), invasive lobular carcinoma in 2 patients (3.9%), and mixed invasive ductal and lobular carcinoma in 1 patient (1.96%). Additional cancers beyond the index cancer were detected in specimens from 8 patients, though these were not included in the analysis.

**Figure 1:**
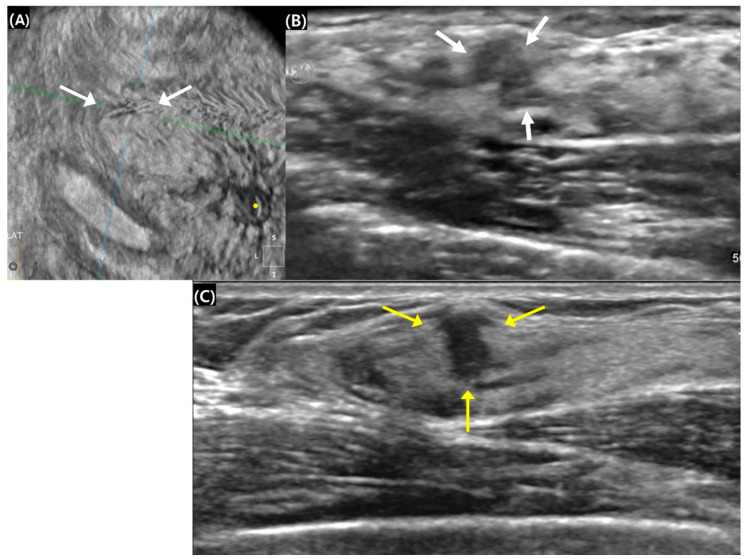
Surgical histopathology revealed a 0.9 cm invasive ductal carcinoma in the right breast of a 45-year-old woman. (**A**,**B**) The 0.8 cm mass with an indistinct margin exhibited isoechogenicity on automated breast ultrasound (white arrows). The green and blue lines served as crossing directional lines to indicate the position and direction of the lesion, and the yellow dot represents the nipple location in (A). (**C**) The mass appeared to have a 0.9 cm spiculated margin with hypoechogenicity on handheld breast ultrasound (yellow arrows). The lesion’s width was 0.5 cm, and the height–width ratio was calculated to be 1.8.

A comparison of US findings between ABUS and HHUS is summarized in Table 1. All lesions evaluated with ABUS were classified as having an irregular shape, resulting in no variability for the statistical analysis of ABUS–HHUS agreement. In contrast, 96.8% of lesions assessed by HHUS were categorized as irregular, further limiting the variability between the two modalities. Regarding margin characteristics, ABUS demonstrated a tendency to show less aggressive margins compared to HHUS, with 61.3% of lesions showing agreement between the two modalities, while 25.8% exhibited fewer suspicious margins on ABUS (Figure 1). However, this difference was not statistically significant (*p* = 0.221). Regarding the height–width ratio, ABUS showed a slightly higher median value (0.98, interquartile range [IQR]: 0.72–1.12) compared to HHUS (0.86, IQR: 0.74–1.10), although this difference was also not statistically significant (*p* = 0.166). Additionally, there were no significant differences between ABUS and HHUS in terms of echo patterns and posterior features (*p* > 0.05), indicating comparable performance in these aspects.

Table 2 presents the analysis of mass margins according to the BI-RADS lexicon, categorizing margins as circumscribed, microlobulated, indistinct/angular, or spiculated, with each classification representing increasing suspicion for malignancy. The overall agreement between ABUS and HHUS was 61.3% (31 of 51 masses), with both modalities showing consistent findings in many cases. However, notable discrepancies were observed: seven cases (12.9%) had more suspicious margins on ABUS compared to HHUS. Specifically, five lesions that had indistinct/angular margins on HHUS were classified as spiculated on ABUS, and two lesions with microlobulated margins on HHUS were reclassified as indistinct/angular on ABUS. On the other hand, 13 cases (25.8%) demonstrated less suspicious margins on ABUS. In these cases, 11 lesions initially assessed as spiculated on HHUS were downgraded to indistinct/angular on ABUS, while 2 lesions with indistinct/angular margins on HHUS were reclassified as microlobulated on ABUS.

**Figure 2:**
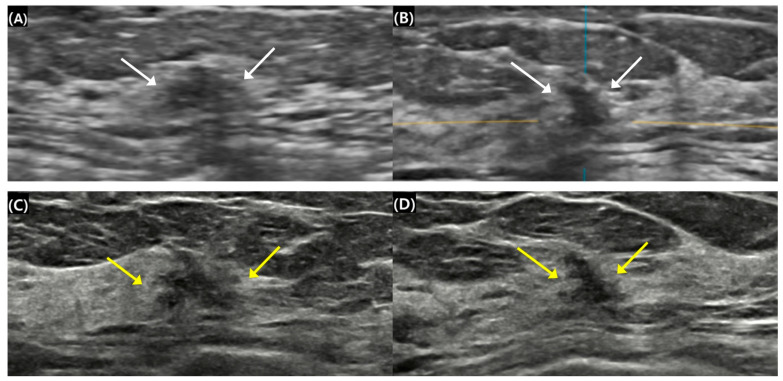
Surgical histopathology revealed a 1.1 cm invasive ductal carcinoma in the left breast of a 52-year-old woman. (**A**,**B**) A 1.0 cm irregular isoechoic mass was categorized as breast imaging reporting and data system (BI-RADS) 4B on automated breast ultrasound (ABUS) (white arrows). The blue and yellow lines served as crossing directional lines to indicate the position and direction of the lesion in (B). (**C**,**D**) The mass was measured at 1.1 cm and showed more heterogeneous echogenicity. The assessment was upgraded to BI-RADS 4C on handheld breast ultrasound (HHUS) (yellow arrows). The lesion received a lower category rating on ABUS compared to HHUS. Size measurements of small breast masses were similar across both ABUS and HHUS modalities. The maximal diameters were 0.92 ± 0.30 cm for ABUS and 0.93 ± 0.29 cm for HHUS (mean ± standard deviation). No significant difference was observed between the two modalities in assessing the size of small breast masses (*p* > 0.05). Pathological results revealed a mean cancer size of 1.10 ± 0.42 cm. Tumor size assessments across ABUS, HHUS, and pathological reports were also comparable, with respective sizes of 0.92 ± 0.30 cm, 0.93 ± 0.29 cm, and 1.10 ± 0.42 cm, showing no significant differences (*p* > 0.05).

The only statistically significant difference between ABUS and HHUS was found in the final BI-RADS assessment categories based on US findings (Table 3). Cross-tabulation of the re-assigned BI-RADS categories revealed that 54.9% (28/51) of the masses had identical classifications between ABUS and HHUS. However, ABUS assigned a lower BI-RADS category than HHUS in 37.3% (19/51) of cases (*p* = 0.005), while still maintaining a level of suspicion for malignancy by classifying these masses as BI-RADS 4A or higher (Figure 2). No masses were categorized as BI-RADS 3 or lower on ABUS, indicating that these lesions were considered likely benign even by ABUS. In contrast, four cases (7.8%) were classified with a higher BI-RADS category on ABUS than on HHUS.

## 4. Discussion

To our knowledge, this study is the first to compare the morphological characteristics, BI-RADS category assessments, and pathological size between ABUS and HHUS in small malignant breast masses (≤1 cm). Our findings demonstrated that ABUS tended to assign lower BI-RADS categories compared to HHUS, with a significant number of lesions being downcategorized, while maintaining malignancy suspicion within categories 4A or higher.

Both ABUS and HHUS are US modalities that provide rapid, radiation-free imaging. Various studies have validated the reliability of ABUS, consistently reporting overall results comparable to those of HHUS [10,21]. In women with dense breast tissue, classified as heterogeneous or extremely dense, combining mammography with screening US significantly enhances the detection of node-negative invasive breast cancer. Incremental cancer detection rates associated with both ABUS and HHUS average between 2.1 and 2.7 per 1000 examinations [22]. Although Wang et al. [23] indicated the superior sensitivity of ABUS for lesions < 1 cm, it has shown a tendency to identify smaller lesions less frequently and to assign them lower final assessment categories. Nevertheless, ABUS has successfully detected all malignant lesions identified on HHUS [11]. Due to these characteristics, ABUS is widely accepted as an effective screening tool, helping to reduce false positives while enhancing breast cancer detection.

In our study, ABUS demonstrated fewer suspicious margins for small breast masses compared to HHUS and was assigned lower BI-RADS assessment categories. We hypothesize that these findings may be influenced by the less frequent identification of small and benign lesions. Choi et al. [9] demonstrated that non-circumscribed mass margins independently correlate with malignancy on ABUS. In particular, spiculated margins exhibited the highest odds ratio for differentially diagnosing breast lesions in both modalities. Additionally, an irregular shape was recognized as an independent predictor of breast cancer in both ABUS and HHUS, aligning with previous investigations that utilized the fourth and fifth editions of the BI-RADS lexicon for HHUS assessment [24,25].

The height–width ratio, while an objective measure, could be influenced by differences between ABUS and HHUS. Factors such as imaging plane selection, manual compression in HHUS, operator variability, and transducer resolution may all affect this ratio. ABUS employs a wide transducer, approximately 15 cm, that applies uniform pressure across the entire breast. This design provides consistent imaging but makes it challenging to achieve focused compression on small breast cancers, particularly when located at the periphery of the scanning field. In contrast, HHUS uses a narrower transducer, typically less than 5 cm wide, allowing targeted compression on the index cancer. Notably, ABUS compresses a larger area simultaneously, whereas HHUS focuses on the lesion site specifically. This discrepancy in compression techniques may explain why ABUS tends to demonstrate a higher height–width ratio compared to HHUS in our study.

Distinguishing characteristics in very small masses can be particularly challenging. Larger masses typically exhibit features that are easier to identify according to the BI-RADS lexicon, while small masses require more meticulous assessment. Variations in US compression techniques may unveil different features, highlighting the importance of understanding how these differences manifest across various methods to ensure that small breast cancers are not overlooked during screening.

When a small breast mass with an irregular shape is detected solely on ABUS, caution is warranted, even if the spiculated margin is not distinctly defined and falls under the category of non-circumscribed margins. Given that even subtle suspicious findings may indicate the presence of cancer, it is advisable to actively consider further evaluation with targeted HHUS and to pursue biopsy rather than defaulting to follow-up. This proactive approach can significantly reduce the risk of false-negative results and enhance the early detection of breast cancer.

The role of ABUS as an adjunctive breast cancer screening tool for mammography has gained increasing recognition in multicenter clinical studies [6,7]. Its utility in patients without clinically and mammographically suspected enlarged axillary lymph nodes is worthy of further consideration. Several studies have reported image–pathology correlations for lesions observed on HHUS [26,27,28,29,30,31], and radiologists are familiar with these findings. However, the correlation between lesions identified by ABUS and pathology remains inadequately established. Recognizing the results of our study, it is essential to note that performing targeted HHUS for lesions identified by ABUS, unless they are typical benign lesions, may lead to a potential upgrade in BI-RADS categorization.

In a previous study utilizing two types of breast phantoms [32], size measurements of phantom lesions using ABUS and HHUS matched precisely, irrespective of shape and elasticity. This study also revealed excellent inter- and intra-observer agreements in size measurements between ABUS and HHUS. Consequently, our results, which indicate no significant differences in tumor size assessments across ABUS, HHUS, and pathological reports, may be readily applicable for clinical use.

Our study had several limitations. First, we analyzed the comparative US features of small breast cancers without the assistance or combination of mammography findings. This study was specifically designed to enhance our understanding of cancer detection in screening ABUS. However, it is important to note that US is typically performed in conjunction with mammography in real clinical workflows. Second, all cancers included in this study were mass lesions, possibly due to the small sample size from a single center. Therefore, we could not assess the differences between ABUS and HHUS for non-mass lesion types, particularly those ≤ 1 cm. Third, this study relied solely on grayscale images without incorporating elastography or Doppler imaging in the assessment of HHUS. We posited that this would maintain a consistent setting for comparison with ABUS. Fourth, a potential limitation is that the reviewers were aware the US images were from breast cancer patients, which may have influenced their evaluation of lesion features. However, as both HHUS and ABUS images were analyzed with this prior knowledge, it is unlikely to have affected the comparison between the two modalities. Lastly, our study aimed to investigate how well the BI-RADS lexicon and assessment categories for small cancers aligned between ABUS and HHUS when ABUS was conducted for screening purposes. However, we did not analyze performance metrics such as sensitivity or specificity. We anticipate that future results from a prospective study, which will serve as the population for our research, will provide further insights into the performance of ABUS and HHUS in the preoperative evaluation of early-stage breast cancer.

In conclusion, small breast cancers measuring ≤1 cm exhibited suspicious US characteristics on both ABUS and HHUS and yet were assigned lower BI-RADS assessment categories on ABUS compared to HHUS. Recognizing the discrepancies in US features for small breast cancers between ABUS and HHUS and actively recommending biopsies for even subtle suspicious findings on ABUS can enhance the early detection of breast cancer through screening.

## Figures and Tables

**Table 1:** A Comparison between automated breast ultrasound (ABUS) and handheld breast ultrasound (HHUS).

US Feature	Category	Number (*n*, (%))	Median (Q1, Q3)	*p*-Value
Shape	Agree	49 (96.8)		NA
	ABUS more suspicious	2 (3.2)		
	ABUS less suspicious	0 (0)		
Margin	Agree	31 (61.3)		0.221
	ABUS more suspicious	7 (12.9)		
	ABUS less suspicious	13 (25.8)		
Orientation (H-W ratio)	ABUS		0.98 (0.72, 1.12)	0.166
	HHUS		0.86 (0.74, 1.10)	
Echo Pattern	Agree	35 (67.7)		0.532
	Disagree	16 (32.3)		
Posterior Feature	Agree	49 (96.8)		0.801
	Disagree	2 (3.2)		
BI-RADS Category	Agree	28 (54.8)		0.005
	ABUS more suspicious	4 (7.8)		
	ABUS less suspicious	19 (37.3)		

NA: Not Applicable—All lesions on ABUS were assessed as having an irregular shape; therefore, statistics cannot be extracted. No.: Number of lesions, Q1: first quartile, Q3: third quartile, H-W ratio: height–width ratio.

**Table 2:** Margin of the masses according to the breast imaging reporting and data system: automated breast ultrasound (ABUS) vs. handheld breast ultrasound (HHUS).

Mass Margins (n = 51)					
ABUS	HHUS				
	Circumscribed	Microlobulated	Indistinct/Angular	Spiculated	Total (n, %)
Circumscribed	2	0	0	0	2 (3.9)
Microlobulated	0	0	2	0	2 (3.9)
Indistinct/angular	0	2	8	11	21 (41.2)
Spiculated	0	0	5	21	26 (51.0)
Total (n, %)	2 (3.9)	2 (3.9)	15 (29.4)	32 (62.7)	51

*n*: number of masses.

**Table 3:** Breast imaging reporting and data system final assessment categorization by automated breast ultrasound (ABUS) and handheld breast ultrasound (HHUS).

ABUS	HHUS				
	4A	4B	4C	5	Total (*n*, %)
4A	3	2	2	0	7 (13.7)
4B	0	2	5	0	7 (13.7)
4C	0	0	13	10	23 (45.1)
5	0	0	4	10	14 (27.5)
Total (*n*, %)	3 (5.9)	4 (7.8)	24 (47.1)	20 (39.2)	51

*n*: number of masses.

## Data Availability

The datasets used and/or analyzed during the current study are available from the corresponding author upon reasonable request.
